# Using green infrastructure to improve urban air quality (GI4AQ)

**DOI:** 10.1007/s13280-019-01164-3

**Published:** 2019-03-16

**Authors:** C. Nick Hewitt, Kirsti Ashworth, A. Rob MacKenzie

**Affiliations:** 1 Lancaster Environment Centre, Lancaster University, Lancaster, LA1 4YQ UK; 2 Birmingham Institute for Forest Research and School of Geography, Earth and Environmental Sciences, University of Birmingham, Birmingham, B15 2TT UK

**Keywords:** Air pollution, Air quality, Green infrastructure, Urban environment

## Abstract

As evidence for the devastating impacts of air pollution on human health continues to increase, improving urban air quality has become one of the most pressing tasks facing policy makers world-wide. Increasingly, and very often on the basis of conflicting and/or weak evidence, the introduction of green infrastructure (GI) is seen as a win–win solution to urban air pollution, reducing ground-level concentrations without imposing restrictions on traffic and other polluting activities. The impact of GI on air quality is highly context dependent, with models suggesting that GI can improve urban air quality in some situations, but be ineffective or even detrimental in others. Here we set out a novel conceptual framework explaining how and where GI can improve air quality, and offer six specific policy interventions, underpinned by research, that will always allow GI to improve air quality. We call GI with unambiguous benefits for air quality GI4AQ. However, GI4AQ will always be a third-order option for mitigating air pollution, after *reducing* emissions and *extending* the distance between sources and receptors.

## Introduction: Urban air quality and green infrastructure

More than half of the world’s population currently live in urban areas, most of which have outdoor air quality that fails to meet World Health Organisation guidelines for healthy living. Air pollution, principally caused by nitrogen dioxide (NO_2_) and fine particles of aerodynamic diameter less that 2.5 µm (PM_2.5_), is now the leading environmental cause of mortality world-wide, causing ~ 3 million premature deaths a year, twice the number due to road traffic accidents (World Health Organisation 2016). While reducing pollutant emissions is always the most direct way to improve urban air quality, authorities world-wide have, with few exceptions, struggled to provide adequate air quality improvements through emission control strategies alone. Policy makers are increasingly turning to complementary methods of reducing human exposure to air pollutants as cities expand, the number of motor vehicles grows (globally from < 0.1 × 10^9^ in 1960 to > 1 × 10^9^ in 2017), and distances driven increase. The relative growth in diesel vehicle numbers, many of which are not compliant with emission regulations (Schiermeier 2015), is an important additional adverse factor in some countries, including the UK.

One increasingly promoted method for air pollution mitigation is the use of green infrastructure (GI): street and park trees, green walls, green roofs (Berardi et al. 2013), and other means of introducing vegetation into the urban landscape (Beatley 2016), on the basis that pollutants deposit more efficiently onto vegetation than onto smoother, impervious, artificial surfaces (Fowler et al. 2009; Nowak et al. 2013; Neft et al. 2016). However, the empirical evidence for the effectiveness of GI for air quality is weak. Without a method to systematically assess GI impacts on urban air, it will remain difficult for researchers and practitioners to determine how and where GI can improve air quality. In offering such a method here, we recognise that known modelling deficiencies and lack of ground-truthing field observations limit the precise quantitative assessment of specific GI interventions. Whereas previous reviews of this topic have focussed on one aspect of the problem (e.g. removal of particles; Janhäll 2015) or have been rather unselective (e.g. Abhijith et al. 2017), here we critically appraise the evidence for the effectiveness of GI in a conceptual framework and offer six specific policy interventions that can only benefit air quality.

GI is part of the urban canopy, set within, and contributing to, its heterogeneity. The character of the urban canyon adds complexity but also offers opportunities to identify sites where GI will have unambiguous benefits for air quality (which we call GI4AQ). Below, we use ‘canopy’ to refer to the volume-filling effects of buildings and trees; we use ‘crown’ when discussing individual tree tops. Metrics describing stem or stand densities do not adequately define the urban tree canopy because of differing tree management methods (e.g. pollarding). Planar cover, while an undoubtedly useful measure (e.g. as used in the *i*-*Tree Canopy* model), leaves the vitally important vertical dimension unconstrained, and neither stem count nor tree-crown cover situates GI three-dimensionally in the urban canyon. Here, we use three underpinning urban canopy-related axioms: that GI will affect air quality most significantly when it (i) fills canopy gaps and edges to alter flow (Oke 1988; Ng and Chau 2012), (ii) alters mean aerodynamic roughness (Barnes et al. 2014; Jeanjean et al. 2015) or (iii) increases the absorbency of surfaces adjacent to polluted air held within the urban canopy (Pugh et al. 2012).

Ground-level concentrations of urban air pollutants are a complex function of emissions, dispersion (stirring and mixing), deposition and chemistry. Much of this complexity is due to the spatial pattern of the urban canopy (Ratti et al. 2006; Abhijith et al. 2017), within which people are exposed to polluted air. The urban canopy occupies near-surface volume (Henderson et al. 2016), interacting with the air flow (Oke 1988). Stirring of parcels of air stretches and folds them, producing irregular blobs and filaments of relatively undiluted emissions interleaved with cleaner air, and mixing dilutes emissions by intermingling them with cleaner air at the molecular scale (Prather and Jaffe 1990; Tan et al. 1998). For urban land-classes dominated by transport corridors (Owen et al. 2006), the landscape is more open with fewer buildings and the canopy largely comprises vegetation (Choi et al. 2014; Abhijith et al. 2017).

Despite the complexities of how urban form impacts the atmospheric concentrations of pollutants, developing a framework around the urban-canopy axioms above can guide policy makers on how and where GI can be used to improve air quality—GI4AQ—and where GI is unhelpful or even detrimental to air quality. Inserting or removing GI with the intention of improving air quality must be considered in the context of other possible co-benefits and costs. For example, urban trees provide habitats that enhance biodiversity, provide shade and other micro-climate services (Livesley et al. 2016; Salmond et al. 2016) and, to a minor extent, sequester carbon dioxide from the atmosphere (Nowak and Crane 2002). Like all urban infrastructure, GI systems, from sophisticated vertical forests (Moeller 2015) to shrubs in planters, require proper installation and regular long-term maintenance to prevent damage to buildings, roads and pavements (Trees, Design and Action Group, TDAG 2012, 2014). Planning with GI should include scenario-based ‘futures thinking’ to ensure long-term efficacy (Lombardi et al. 2012; Hale et al. 2015). For example, trees in street canyons which currently reduce dispersion of traffic pollutants (see below) may be less of a concern in the future when electric or hydrogen vehicles will cause much less street-level pollution emissions (Jacobson et al. 2005). Likewise, in the past, when major pollution sources were mainly situated above roof level, the impact of street trees on pollutant dispersion within the street canyon was not a significant concern.

A useful conceptualisation of air pollution mitigation in urban areas is “Reduce–Extend–Protect”. *Reducing* emissions is always the most effective method of reducing human exposure to pollutants and should always be the primary focus of mitigation action. GI does not play any explicit role in this. *Extending* the distance between sources and receptors, enhancing dilution and dispersion and hence reducing concentrations at a given receptor, is usually the second-best method of reducing exposure. This may be done by physically extending the distance between, for example, road vehicles and pedestrians, or by placing barriers to flow between sources and receptors. GI can act in this role, for example when hedges are used to separate traffic and pedestrians, virtually *extending* the distance between source and receptor. *Protecting* receptors involves introducing direct interventions that reduce concentrations at the receptor site, and here GI can be used in several configurations, as discussed below. This will normally be the third-best mitigation option.

## Dispersion of air pollutants

Trees and hedges provide semi-permeable obstacles to the flow of air (Bradley and Mulhearn 1983; Raine and Stevenson 1977; Tiwary et al. 2005; Gromke et al. 2016; Tong et al. 2016), deflecting stream-lines, introducing turbulence and increasing dilution and hence virtually *extending* the distance between source and receptor. Several structural factors, such as plant height and morphology, affect the way vegetation interacts with flow, and can be considered design parameters (Baldauf 2017) for GI4AQ. Dense vegetation acts almost as a bluff body, with negligible permeating flow and a region of recirculation behind the vegetation (Tiwary et al. 2005). For crown porosities above ~ 50%, no recirculating region forms behind the obstacle (Baltaxe 1967; Bradley and Mulhearn 1983). Porosities of common urban GI4AQ are listed in a recent review (Abhijith et al. 2017).

**Fig. 1:**
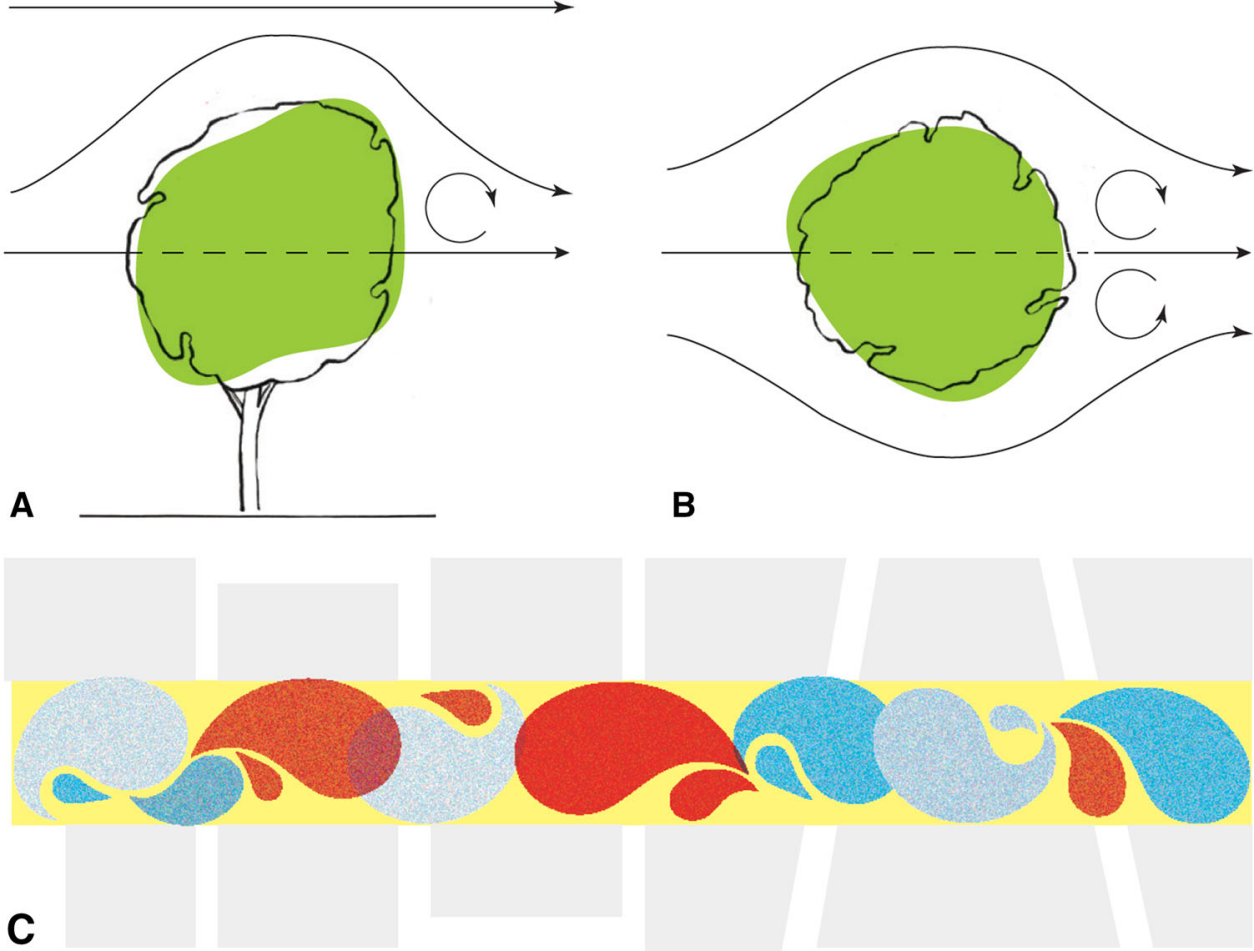
Schematic representation of flow around a dense tree crown, **a** in elevation and **b** in plan, and **c** street trees can cause areas of relatively lower (blue) and higher (red) ground-level pollutant concentrations, with the street-average concentration shown in yellow (adapted from Jeanjean et al. 2017). In the plan view cartoon of a street canyon containing trees (**c**), the trees will be approximately located at the intersections of the red and blue filaments of air with higher and lower pollutant concentrations, initiating disturbances in the down-wind flow at these points

Regions of accelerating and decelerating air stir pollutants into filamentary patches of higher and lower concentrations (Gromke and Blocken 2015) (Fig. 1). Resolving these spatial variations at the street scale requires resource-intensive computational fluid dynamics (CFD) modelling, supported by site-specific crown and canopy measurements (Hofman et al. 2016). The modelled aerodynamic effect of street trees for two main roads in London, for example, was quasi-two dimensional, and reductions in the average concentrations in the street canyons were negligible (1%) (Jeanjean et al. 2017a, b). Under other circumstances, canopy-induced turbulence in the model led to three-dimensional stirring and mixing, reducing average ground-level concentrations (Barnes et al. 2014). Modelling using a remotely sensed inventory of tree-top pattern calculated a median reduction of 8% in ground-level concentrations of PM_2.5_ across a specific city centre due to the dispersive effect of the trees present (Jeanjean et al. 2015). In contrast, a recent summary reported increases of between 0 and 96% in modelled average street canyon pollutant concentrations due to the introduction of trees (Abhijith et al. 2017), highlighting both the uncertainties in current models and the need for caution when introducing trees to street canyons.

Contiguous and dense tree crowns can effectively separate the air below the canopy from that above (Gromke and Blocken 2015). A reversal of flow at 2 m above street level for street trees spaced at 25-m intervals (Moradpour et al. 2017) dramatically exemplifies such behaviour in models. In parks, traffic-free plazas, and other pedestrian areas without significant ground-level anthropogenic pollution sources, but with dense vegetation canopies, the below-canopy air will always be cleaner than that above the canopy due to enhanced deposition of pollution onto the vegetation as the air percolates through the canopy (see below). However, when canopy closure occurs in a street canyon containing ground-level sources of pollution, pollutants may be trapped, leading to increased ground-level concentrations (Vos et al. 2013; Abhijith et al. 2017). In such situations, local emission controls should be implemented to reduce or remove the ground-level pollution source. When emissions cannot be adequately reduced, it is necessary to identify which elements of the urban canopy are inhibiting vertical mixing and, hence, what modifications to the canopy (including tree crowns) can be made to improve ventilation and so improve ground-level air quality (GI4AQ Policy Intervention 1, see Table 1). CFD studies provide the only quantitative method currently available to quantify ventilation, but many such studies do not capture the intermittency of turbulent flow and all lack field observations for model evaluation.

**Table 1:** Summary of GI4AQ Policy Interventions (PIs)

PI1	Carry out modelling (probably using computational fluid dynamics) to identify causes of reduced ventilation in streets with closed tree canopies where emission reductions have not been sufficient to achieve acceptable air quality. Modify canopy to increase street canyon ventilation accordingly
PI2	Introduce hedges (and other linear barriers) between traffic and pedestrians. Choose barrier height, porosity and length to maximise benefits. This may require dispersion or computational fluid dynamics modelling
PI3	Provide long-term effective management of GI to ensure continuation and maximisation of the ecosystem service of enhanced pollutant deposition
PI4	Introduce and maximise areas of green walls in street canyons
PI5	Create “green oases”, i.e. slowly ventilated zones containing or surrounded by GI but with no internal pollution sources. Green oases may range in size from a bench closely surrounded by high hedges to a city park with a dense tree canopy
PI6	When planning to increase or change the urban tree population by more than ~ 10% at the city-wide scale, assess the impact on ground-level ozone and choose low VOC-emitting tree species to minimise any increases in down-wind ozone pollution

**Fig. 2:**
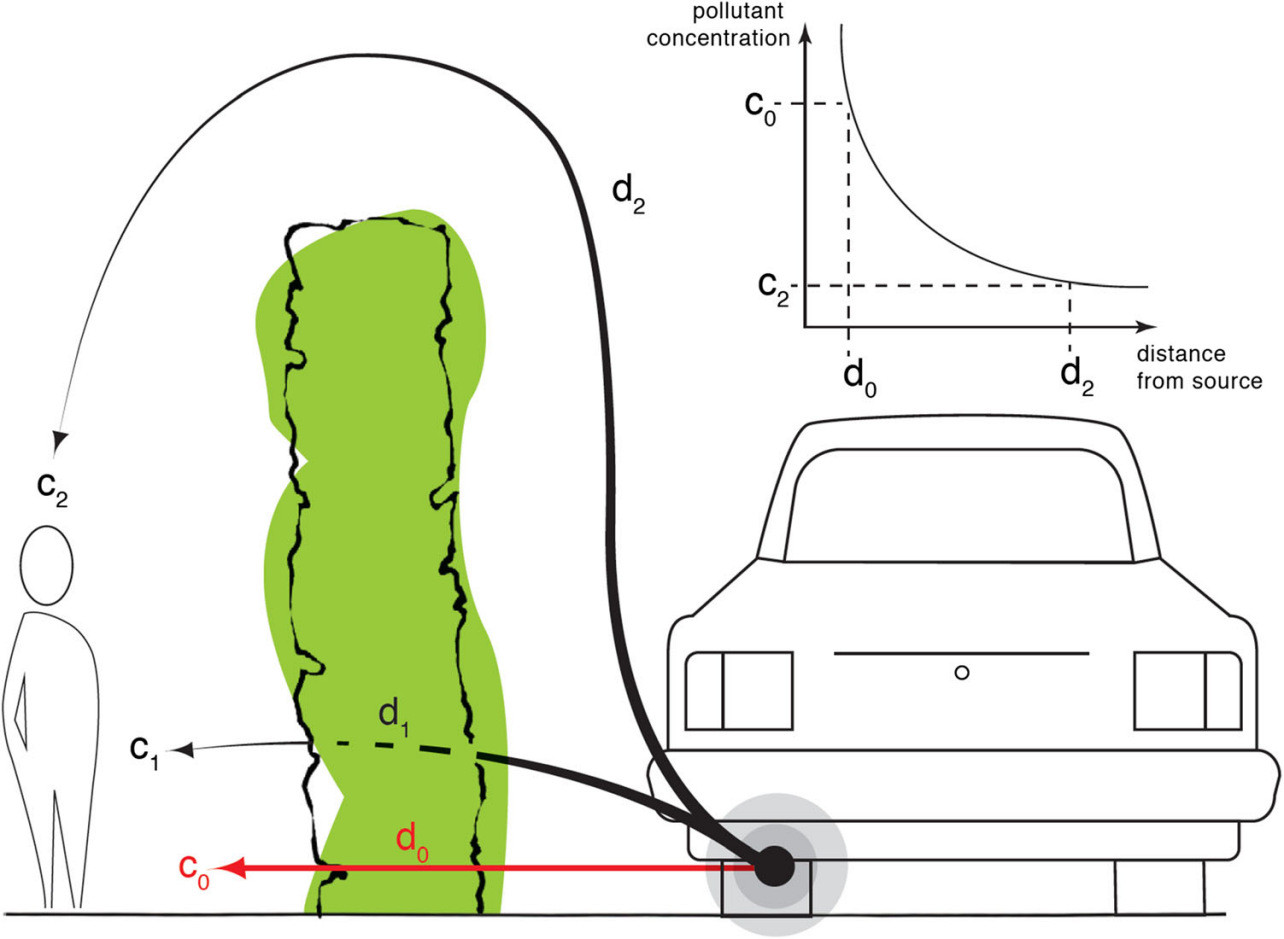
Effect of a permeable linear barrier or hedge on pollutant concentrations. The pollutant concentration experienced by the child receptor is the mass-weighted average of the concentrations through (*c*_1_) and over (*c*_2_) the linear barrier. Along paths *d*_1_ and *d*_2_, pollutant concentrations are diluted by mixing and deposition. Deposition dominates for *d*_1_, mixing dominates for *d*_2_, with *c*_2_ decreasing approximately exponentially (see inset). The characteristic mixing length-scale is determined by local turbulence. In the absence of the linear barrier, the receptor experiences higher concentration, *c*_0_, diluted over shorter distance, *d*_0_, and not subject to enhanced deposition to vegetation

Dispersion will always ultimately transfer pollutants down concentration gradients into the cleaner atmosphere or towards absorptive surfaces. As pollutants move from their source, turbulence dilutes the plume by mixing in cleaner air, as recognised in operational air quality models (e.g. Heist et al. 2013; Stocker et al. 2013; Design Manual for Roads and Bridges 2017) and more sophisticated simulations (Tong et al. 2016). The introduction of linear obstacles (e.g. hedges or fences) between source and receptor zones displaces the pollutant plume upwards (Bowker et al. 2007), *extending* the effective path-length of air from source to receptor, and may also promote dilution by enhancing turbulence. Hence hedges and fences can reduce concentrations along pavements, side-walks and other pedestrian areas adjacent to traffic (Gallagher et al. 2015; Gromke et al. 2016; Abhijith et al. 2017) (Fig. 2). Decreases in pollution concentrations of 20–70% (average 52%) behind a 1-m-high impermeable barrier in an open setting have been modelled (King et al. 2009). The effect of barriers on concentrations is complicated by street-scale circulations within a street canyon (McNabola et al. 2009; Gromke et al. 2016; Abhijith et al. 2017).

As the porosity of the barrier increases, the effective path-length decreases (Fig. 2) but the opportunity for removal of particles by deposition increases (Tong et al. 2016). The collection efficiency for a 2.2-m-high, 1.6-m-wide porous hawthorn hedge was measured at ~ 1% for particles < 2.5 µm diameter, increasing to ~ 30% for particles of 15 µm diameter. These results could be reproduced adequately using 2D modelling with appropriate treatment of drag and particle collection (Tiwary et al. 2005; Guo and Maghirang 2012). In general, linear barriers are helpful in aiding dispersion and deposition and hedges (and fences) may therefore offer some protection to pedestrians (GI4AQ Policy Intervention 2). Such obstacles need not be GI (Gallagher et al. 2015), although porous GI or a mix of hard barrier and GI (Tong et al. 2016) would offer co-benefits through enhanced deposition of both large (diameter, *d* > 1 µm) and small (*d* < 100 nm) particles (Neft et al. 2016).

## Deposition of air pollutants

In contrast to dispersion, the deposition of a pollutant to a surface results in permanent loss from the atmosphere, and hence a reduction in total atmospheric loading. Wet deposition is associated with precipitation and proceeds at the same rate to all surfaces (Sehmel 1980; Fowler et al. 2004). However, the rate of dry deposition is highly dependent on the macroscopic characteristics of the surface, i.e. available surface area (Padro 1996; Fowler et al. 2004; Gröte et al. 2016) and surface aerodynamic roughness (Sehmel 1980), and GI can potentially *protect* against air pollution by enhancing the deposition rates of pollutants and hence reduce concentrations of pollutants in the vicinity of receptors. The mix of plant species and size used in GI, and their spatial relationship to the built environment, will determine these deposition parameters and, hence, determine the maximum potential rate of pollutant dry deposition. Particle deposition velocities as high as 11 mm s^−1^ have been measured to urban trees, compared with around 3 mm s^−1^ to adjacent grass, and dry deposition has been estimated to account for ~ 70% of total deposition to urban trees compared with ~ 25% to grass (Fowler et al. 2004).

Vegetation with higher surface area, greater rates of transpiration, and longer in-leaf periods result in the greatest enhancements in dry deposition over that to bare surfaces (Padro 1996; Branford et al. 2004; Nowak et al. 2006; Cabaraban et al. 2013; Gröte et al. 2016). For this reason, the selection of species is critical in determining the increased pollutant removal achieved through the addition of GI to the built environment. For example, the available surface area of deciduous broad-leaved trees can reach up to 6 m^2^ per m^2^ of bare ground (Nowak et al. 2006), 20% more than evergreen needle-leaf trees (van den Hurk et al. 2003). Leaf and plant morphology also contribute to the overall rate of dry deposition to different vegetation species and should be considered in combination with surface area (Gröte et al. 2016).

On-line tools have been developed to assist in species selection (e.g. i-Trees Species Selector 2017 and the derivative European Specifind 2017) but these are of necessity black-box database search instruments giving a list of potential species that acts as a starting point for refinement against other considerations. These on-line tools usually assume optimal physiological behaviour of the GI, but poor soils, high temperatures exacerbated by the urban heat island effect and limited water availability often combine to reduce leaf area and transpiration, reducing deposition rates to well below that for unstressed vegetation (Calfapietra et al. 2015). Effective management of GI (Lu et al. 2010; Young 2011; Pincetl et al. 2013), e.g. to avoid water stress, is therefore essential to ensure its long-term health and functioning and to maximise deposition rates (GI4AQ Policy Intervention 3).

In addition to plant morphology, the characteristics of the canopy play an important role in modifying surface roughness and turbulence. There is the potential to design heterogeneity into the urban canopy to exploit edge effects and maximise deposition. Particle removal by dense forest canopies has been observed to be over 30% higher than to adjacent open heathland with the greatest increases (over 50%) occurring at the forest edge (Branford et al. 2004).

As the rate of dry deposition is proportional to the local concentration of the pollutant (for a given surface and wind flow), GI is most effective at improving air quality in locations where pollutant concentrations are highest (Nowak et al. 2006; Morani et al. 2011; Cabaraban et al. 2013) and where residence times are longest (Pugh et al. 2012). Where GI acts on large volumes of air, for example in the case of green roofs upwind of street canyons, where there will not be a shallow boundary layer or constrained volume of air above the roof surface, the potential to reduce atmospheric concentrations of pollutants is very limited (typically < 1%) (Donovan et al. 2005; Pugh et al. 2012). The capital and maintenance cost of green roofs is therefore likely to be a very poor investment for air quality mitigation.

Measuring or modelling the potential mass of pollution deposited for given air concentrations can make the GI4AQ effect appear to be significant (Nowak et al. 2006; Speak et al. 2012; Berardi et al. 2013), but calculations or measurements of deposition should be combined with modelling of resultant changes in atmospheric concentrations to properly estimate the actual air quality benefits of GI4AQ (Hofman et al. 2016). Recent developments in the application of eddy covariance methods for measuring deposition rates of pollutants offer the possibility of model validation, although probably at only a relatively large (urban park) scale (Guidolotti et al. 2017). In fact, increasing deposition rates will often not result in discernible reductions in atmospheric concentrations, but where GI acts on relatively small volumes of air and ventilation rates are relatively low, models predict that the effects on ground-level air quality can be very large (Pugh et al. 2012). For this reason, the introduction of large areas of green walls in street canyons may be particularly effective at improving ground-level air quality (GI4AQ Policy Intervention 4).

Creating “green oases”, i.e. slowly ventilated areas containing or surrounded by GI but with no internal anthropogenic pollutant sources, will always lead to an improvement in air quality. Green oases can vary in scale from a bench or other small areas surrounded by relatively tall GI, e.g. hedges, up to pedestrianised and verdant street canyons, plazas or courtyards, or even to a park covered in an extensive vegetated trellis roof. In these cases, the amount of GI present should be maximised (GI4AQ Policy Intervention 5).

## Negative impacts of trees on air quality through effects on atmospheric chemistry

All plants synthesise reactive volatile organic compounds (biogenic VOCs) and emit them to the atmosphere. The single most important bVOC by emitted mass and reactivity is isoprene (C_5_H_8_, 2-methyl-1,3-butadiene) but several tens of other bVOCs have significant effects in the atmosphere (Atkinson and Arey 2003; Guenther et al. 2012). As well as these constitutive emissions, biotic and abiotic stresses may induce the production of many other compounds (Hatanaka 1993). For an overview of bVOC synthesis pathways, their biological functions and their emissions and effects in the atmosphere, see Laothawornkitkul et al. (2009).

Although the vast majority of VOCs emitted globally are biogenic in origin (Guenther et al. 1995, 2012), emissions from anthropogenic sources are relatively much more important in urban areas. Nevertheless, isoprene, which has both biogenic and anthropogenic sources, may still be important in urban areas, especially in summer (e.g. Wang et al. 2013), even in temperate cities such as London (Langford et al. 2010).

In the context of urban GI, the most significant bVOC emissions are those from trees, since in almost all urban situations trees will contribute the majority of leaf biomass. Constitutive emissions vary considerably in chemical composition between tree species. Urban areas may contain a large number of tree species, as native species will often be augmented by a wide range of exotics, especially in parks and gardens, all with differing bVOC emission profiles and rates. For example, 126 different species of mature trees have been recorded in London (Treeconomics 2015) and 170 in Beijing (Yang et al. 2005).

bVOCs take part in chemical reactions in the atmosphere that can lead to the formation of ozone (MacKenzie et al. 1991; Chameides et al. 1988; Atkinson and Arey 2003; Donovan et al. 2005; Calfapietra et al. 2013) and organic aerosol particles (Carlton et al. 2009; Hallquist et al. 2009; Mentel et al. 2009; Wyche et al. 2014), both of which are important secondary air pollutants. Since it takes several hours before these chemical reactions generate high pollutant concentrations of ozone or particles, the precise location of bVOC-emitting GI within the urban canopy is not important. This is in contrast to the dispersion and deposition effects of GI, which are highly location-specific. From a policy perspective then, when GI is being implemented for pollution control by dispersion and deposition, the negative effects on secondary air pollution (i.e. ozone and particle formation) can be considered separately, at the urban air-shed, rather than the local, scale.

bVOC emissions from a typical urban tree population contribute on the order of 10% to ozone concentrations within and downwind of large city-regions (MacKenzie et al. 1991; Chameides et al. 1988; Donovan et al. 2005; Calfapietra et al. 2013). Unfortunately, there is no easy way to reliably predict whether or not a given tree species emits a particular bVOC, or at what specific rate. Notwithstanding this, if the total urban tree population is to be altered significantly, e.g. by more than ~ 10%, care should be given to the choice of tree species used, in order to not exacerbate the bVOC emission rates at the urban air-shed scale (GI4AQ Policy Intervention 6). Several (incomplete and largely uncritical) bVOC emission databases (http://www.es.lancs.ac.uk/cnhgroup/iso-emissions.pdf; Keenan et al. 2009; http://bai.acom.ucar.edu/Data/BVOC) may be referred to when selecting tree species for planting, based on their likely bVOC emissions. A more sophisticated assessment might weigh deposition benefits against secondary pollutant formation potentials for individual tree species, to generate, for example, an “Urban Tree Air Quality Score” (Donovan et al. 2005).

Two policy-relevant implications arise from the fact that trees take decades to mature, with bVOC emissions increasing as their leaf area increases over time. First, in the next few decades there is the possibility that urban transport will become less polluting than currently, leading to lower secondary pollutant formation. Ozone isopleths, or ‘Sillman plots’ (1999), which relate ozone pollution to NO_*x*_ and VOC emissions, can be used to estimate the emission reductions from traffic needed to ensure that any additional bVOC emissions resulting from tree planting do not produce additional ozone. Secondly, climate change will lead to increased temperatures, especially in urban areas (Fowler et al. 2008; Estrada et al. 2017), increasing bVOC emissions and therefore exacerbating ozone pollution events (Yang et al. 2008), enhancing the relevance of Policy Intervention 6.

### Policy guidance and conclusions

Numerous modelling studies suggest it is possible to make GI interventions that will improve urban air quality, but there is little unequivocal empirical evidence or validation to support this, although this may change as new measurement technologies become available (e.g. Guidolotti et al. 2017). In situations where pollutant concentrations change rapidly in space and time (e.g. near to roads), measuring small changes in concentrations and attributing these to the introduction of GI is almost impossible. Laboratory-scale experiments have limited utility because deposition and dispersion are very tightly coupled to the three-dimensional urban form and the synoptic-scale flow, while designing field-scale experiments involving GI with adequate controls is difficult, if not impossible. Policy makers must therefore make decisions on GI largely based on model predictions rather than empirical evidence. To aid this, we have identified six GI4AQ Policy Interventions, deduced from an understanding of the processes operating in the near-surface urban air volume (Table 1). All these interventions are risk-free in the sense they can only benefit ground-level air quality, although the effectiveness of specific interventions will vary from the insignificant to the highly significant. Effectiveness may be hard to determine empirically. This is in contrast to other possible actions involving GI that may be detrimental to air quality (e.g. introducing trees into a street canyon, which may increase canopy closure and reduce ventilation rates), or those that may have no discernible effects on air quality (e.g. building green roofs).

A common fallacy concerning urban GI is that increasing the amount of vegetation reduces ground-level pollutant concentrations linearly (i.e. that doubling leaf area will half pollutant concentrations). The vegetation deposition sink is at a distance from the pollutant emission source, so atmospheric concentrations will be always a non-zero, positive-definite, balance of emissions, advection, deposition, and reaction. Not accounting for other terms in the budget leads to over-estimation of the efficacy of green roofs and other forms of GI on air quality, to the detriment of rational decision making.

**Fig. 3:**
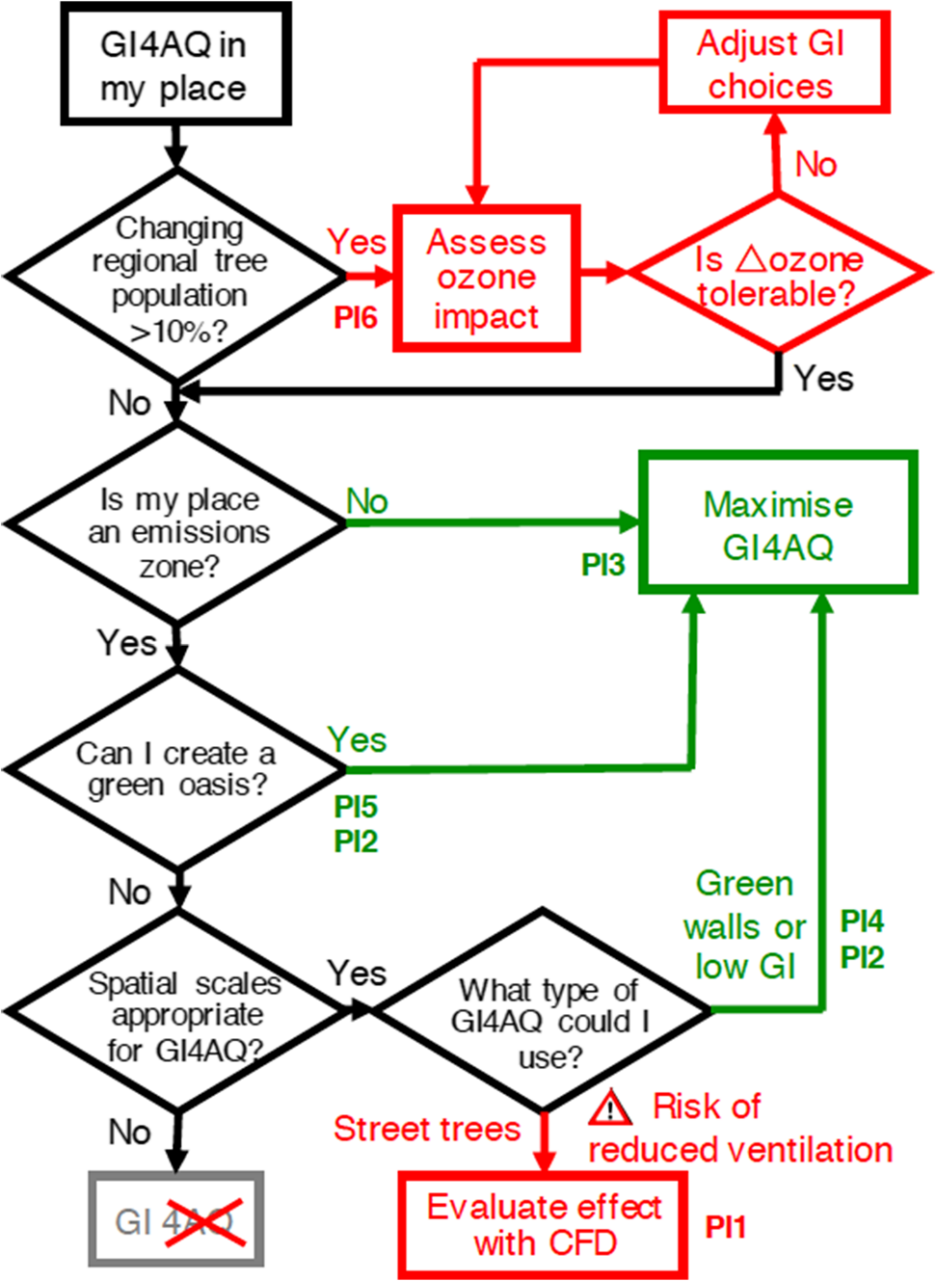
Flow chart to aid GI4AQ decision making. PI1, PI4, PI5 and PI6 refer to the GI4AQ Policy Interventions shown in Table 1. “Regional tree population” refers to the tree population in an area relevant to the production of ground-level ozone from bVOC precursors, i.e. equivalent to several hours travel time of a typical air parcel. “Δozone” is the expected increment to peak ground-level ozone within or downwind of the urban area due to the change in regional tree population. Grey boxes indicate that GI is not suitable for air quality improvements but may provide other ecosystem services. Red boxes require further site-specific measurements and/or modelling before a rational decision can be reached. Capturing evidence used along the paths to a Green box (‘Go’) will improve decision-making transparency and resilience (e.g. Lombardi et al. 2012; Hale et al. 2015). Refer to main text for methods to assess the impact on ozone and for a definition of ‘green oasis’. Appropriate spatial scales for GI4AQ are mapped in Fig. 4

Figure 3 is a flow chart designed to help policy makers navigate the few critical decisions that determine the suitability of GI4AQ—from a scientific perspective—at all relevant spatial scales, from the smallest urban park to a ‘million trees’ Initiative. The flow chart indicates that some policy decisions (marked by green paths in the figure) may be safely reached by the application of simple rules of thumb and the existing literature. Other decisions require specialist and resource-intensive model simulations of dispersion and/or atmospheric chemistry (red paths in the figure) but may still warrant investigation. GI choices shown in grey will be ineffective for air quality improvement but may, of course, still provide other ecosystem services (Beatley 2016). The flowchart should therefore help to prioritise GI interventions when intended for AQ benefits and indicate which GI investment decisions should be supported by more detailed studies.

**Fig. 4:**
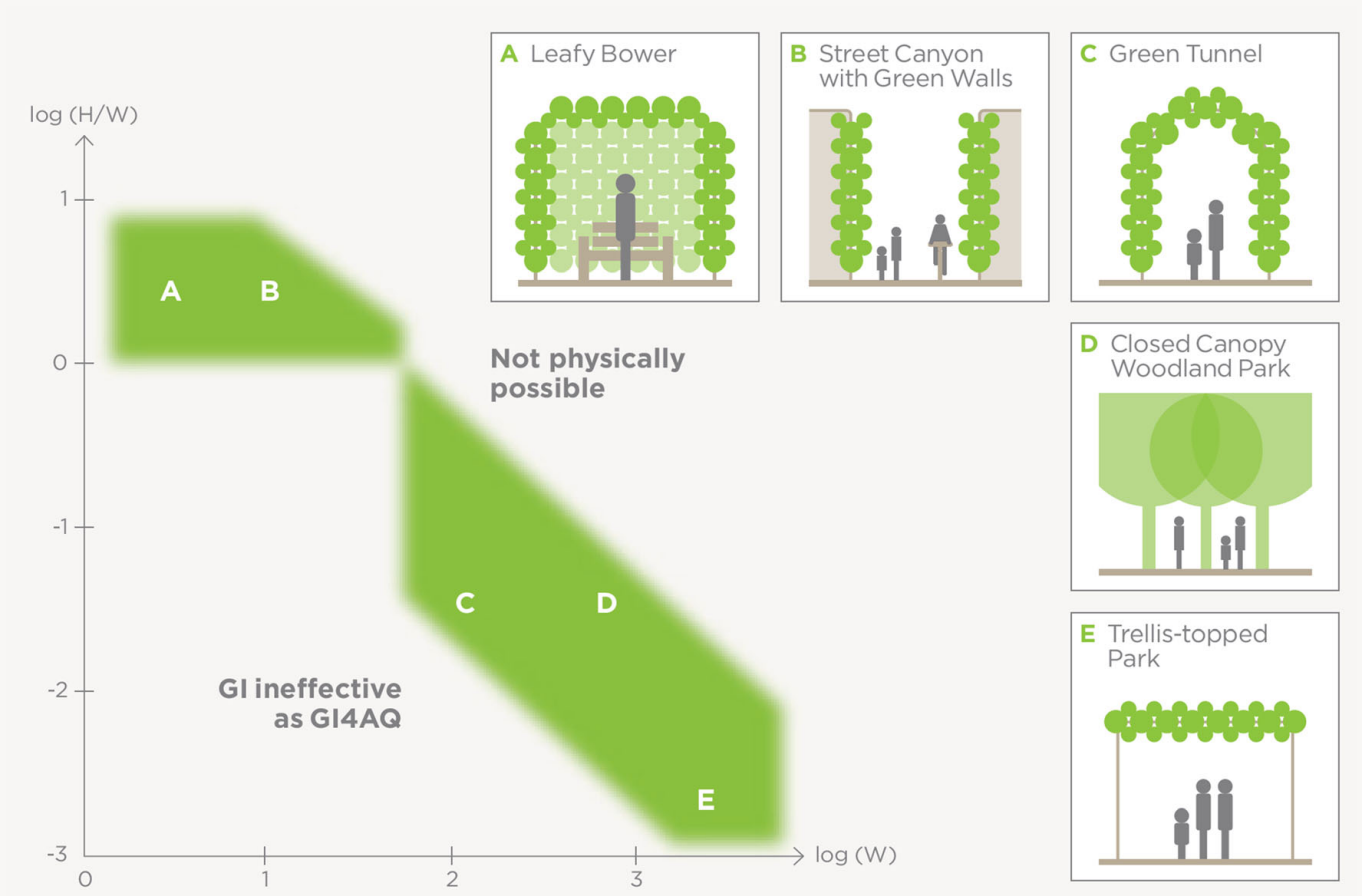
Plot of log(aspect ratio) against log(linear dimension in m), showing space domains in which GI4AQ is feasible and potentially effective. Examples of specific GI4AQ typologies are (from left to right), a bench closely surrounded by high hedges; an extensive green wall in a street canyon, where *W* is the width of the street; a tunnel or canopy of dense vegetation offering protection to pedestrians; a city park with a dense tree canopy. The domain space in the top-right of the figure is physically inaccessible because of limits to the heights of trees and other forms of GI. Green roofs have horizontal scales of tens of metres and *H*/*W* ≪ 1, and so fall in the bottom left corner of the figure, where GI is ineffective for AQ mitigation (see text)

GI4AQ can be effective over a range of horizontal and vertical spatial scales, although there are limitations. It may be helpful to consider an intervention in terms of its characteristic horizontal scale and its height-to-width aspect ratio (Fig. 4). When horizontal length scales and aspect ratios are small, residence times are short and there is little opportunity for deposition to become effective. When aspect ratios are large, especially at large horizontal scales, it becomes physically impossible to manufacture the GI4AQ intervention. GI4AQ is effective where deposition can be enhanced by holding air for longer near vegetation. The space domains in which GI4AQ is likely to be effective range in size from a small “green oasis” such as a bench closely surrounded by high hedges to a dense urban woodland.

Green roofs have horizontal scales up to tens of metres and aspect ratios ≪ 1, and so fall in the bottom left-hand corner of Fig. 4. While they enhance the deposition of pollutants from the atmosphere by increasing the available surface area (Yang et al. 2008; Treeconomics 2015), they are unlikely to make an appreciable difference to ground-level pollutant concentrations since they act on the very large volume of air above the urban canopy (Pugh et al. 2012). Vertical forests (e.g. Moeller 2015) have modest horizontal extent and very large aspect ratios but will be ineffective as GI4AQ because they do not produce either a closed canopy or an open top green oasis. In contrast, green walls in street canyons with aspect ratios greater than about unity [log (*H*/*W*) > 0] may make appreciable differences to ground-level concentrations (Pugh et al. 2012).

Despite the complexities of modern cities, the conceptual framework outlined above, underpinned by research, allows us to provide guidance to policy makers on where and how GI can benefit urban air quality. When proper consideration of context is made, there are clear and substantive opportunities to employ GI to improve air quality. The framework will also help practitioners and policy makers assess new research on GI and air quality as it becomes available. Properly designed and implemented GI4AQ (Lombardi et al. 2012; Trees, Design and Action Group 2014; Beatley 2016) may help cities meet several of the UN’s Sustainable Development Goals, but poorly designed GI may be ineffective or even detrimental to urban air quality. Importantly, decisions on GI4AQ must be made in the wider context of all the costs and benefits of trees (and other GI) in cities (Daniels et al. 2018), for example as one component of a wider “Urban Tree Score” Framework (Donovan et al. 2005).

Finally, it should be noted that the most direct and sure way to improve urban air quality is by *reducing* primary pollutant emissions and the focus of air pollution policies should always be on this. As a secondary measure, it is always beneficial simply to *extend* the distance between sources and receptors at all horizontal scales. Introducing GI4AQ should therefore normally be considered a third-best measure that may, in some situations, help improve urban air quality.
